# Applications of Ni_3_Al Based Intermetallic Alloys—Current Stage and Potential Perceptivities

**DOI:** 10.3390/ma8052537

**Published:** 2015-05-13

**Authors:** Pawel Jozwik, Wojciech Polkowski, Zbigniew Bojar

**Affiliations:** Department of Advanced Materials and Technologies, Faculty of Advanced Technologies and Chemistry, Military University of Technology, Kaliskiego 2 Str., 00-908 Warszawa, Poland; E-Mails: wpolkowski@wat.edu.pl (W.P.); zbojar@wat.edu.pl (Z.B.)

**Keywords:** Ni_3_Al intermetallic alloys, applications, bulk materials, thin foils

## Abstract

The paper presents an overview of current and prospective applications of Ni_3_Al based intermetallic alloys—modern engineering materials with special properties that are potentially useful for both structural and functional purposes. The bulk components manufactured from these materials are intended mainly for forging dies, furnace assembly, turbocharger components, valves, and piston head of internal combustion engines. The Ni_3_Al based alloys produced by a directional solidification are also considered as a material for the fabrication of jet engine turbine blades. Moreover, development of composite materials with Ni_3_Al based alloys as a matrix hardened by, e.g., TiC, ZrO_2_, WC, SiC and graphene, is also reported. Due to special physical and chemical properties; it is expected that these materials in the form of thin foils and strips should make a significant contribution to the production of high tech devices, e.g., Micro Electro-Mechanical Systems (MEMS) or Microtechnology-based Energy and Chemical Systems (MECS); as well as heat exchangers; microreactors; micro-actuators; components of combustion chambers and gasket of rocket and jet engines as well components of high specific strength systems. Additionally, their catalytic properties may find an application in catalytic converters, air purification systems from chemical and biological toxic agents or in a hydrogen “production” by a decomposition of hydrocarbons.

## 1. Introduction

Intermetallics are a unique group of materials composed of two (or more) types of metal (or metal and non-metal) atoms, which exist as solid compounds and differ in a structure from that of the constituent components. In comparison to conventional metals and alloys, intermetallic based alloys exhibit several specific features. Traditional materials are composed of solid solutions of two or more metallic or nonmetallic elements in a metal lattice. In conventional alloys, atoms are bonded by relatively weak metallic bonds. In the case of ordered intermetallics, strong ionic and covalent bonds also exist in a crystalline lattice. Moreover, atoms always take their strict positions in a crystalline lattice—forming a so called ordered superlattice, which is characterized by a long range order (LRO) stable up to a critical temperature of ordering [1,2,3,4,5].

These structural characteristics are responsible for physical and mechanical properties of intermetallics, namely a relatively high melting point, a high strength (especially at elevated temperature) and also a relatively low ductility. These properties make them similar to ceramics. However, unlike ceramic materials, intermetallics exhibit also a metallic shine, a good thermal and electric conductivity, as well as some susceptibility to plastic deformation, which shifts them toward metallic materials [2,3,4,5,6,7,8].

Since a great number of intermetallic phases have been recognized and examined, the most advanced works in the field of metallurgy and performance properties have been carried out for intermetallic phases from the Ni–Al, Fe–Al and Ti–Al binary systems. The main research efforts have been focused on NiAl, Ni_3_Al, FeAl, Fe_3_Al, TiAl, Ti_3_Al and TiAl_3_ based alloys [5,6,9,10,11,12,13,14,15,16,17,18,19,20,21]. These materials have already found industrial applications or are close to a wide commercialization [2,3,4,5,6,7,8,9,10].

The research on the Ni_3_Al intermetallic phase started in the 1940s. At that time, the Ni_3_Al phase was playing a role of the main strengthening component in nickel based superalloys (its volume fraction was below 20%). Unique properties of this phase were a driving force for extensive studies on the Ni_3_Al based alloys, which were terminated after finding an extremely low plasticity of a polycrystalline Ni_3_Al. A breakthrough came in 1979, when Aoki and Izumi [11] discovered an unexpectedly positive impact of a boron addition on the ductility of intermetallics. This finding renewed a scientific and industrial interest in intermetallic phases—with particular emphasis on Ni_3_Al and NiAl. In 1980, Oak Ridge National Laboratory (ORNL) launched a research program on intermetallic alloys, bringing together over than 100 research institutes. As a consequence, a few commercial Ni_3_Al based intermetallic alloys were developed and introduced to various branches of industry.

Nevertheless, it should be emphasized that an industrial potential of the Ni_3_Al alloys has not been fully utilized. Research works on these materials are still carried out, however the main activities are now being shifted toward a development of processing technologies (e.g., that allows producing strips or foils with a nanocrystalline structure, *etc.*) as well as catalytic materials [22,23,24,25,26,27,28,29,30,31,32,33,34,35] and intermetallic matrix composites [36,37,38,39,40].

Available reviews on applications of Ni_3_Al based alloys are limited only to bulk materials [10,19,20,21,41] and the most recent were published in 2000. The authors’ intention is to fill the existed gap in a description of actual and future applications of Ni_3_Al based alloys.

## 2. Properties of Ni_3_Al-Based Alloys

**Figure 1:**
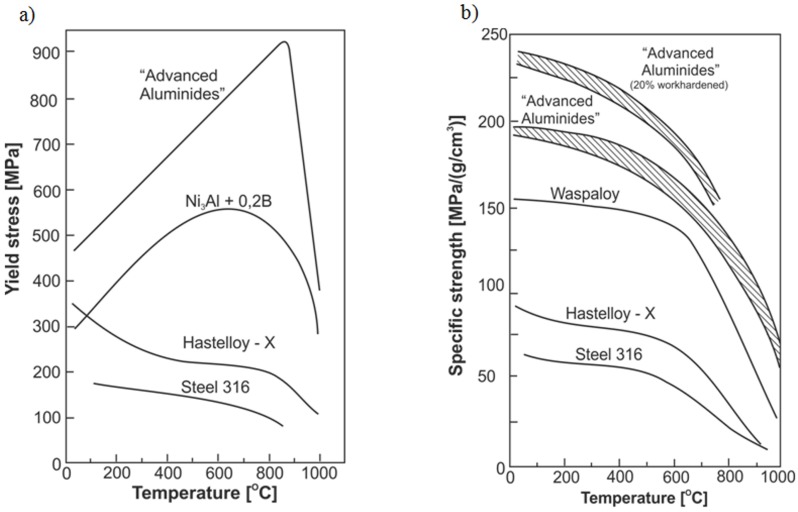
A comparison of the yield strength (**a**) and specific strength (**b**) *vs.* deformation temperature plots for the Ni_3_Al based alloys and conventional heat resistant alloys (Hastelloy X and 316 stainless steel). The term “advanced aluminides” denotes a Ni_3_Al based alloy with an addition of boron and hafnium (based on [43]) (*Courtesy of Oak Ridge National Laboratory, U.S. Department of Energy*).

There are many literature analyses and comparisons of physicochemical and mechanical properties of the Ni_3_Al intermetallic alloys with those of classical metallic materials. The Ni_3_Al alloys are mostly superior to the commercial alloys, especially in the field of high-temperature properties, in an oxidizing and carburizing environments. The most attractive properties of the Ni_3_Al intermetallics include:

- a high tensile and compression strength at temperature of 650 ÷ 1100 °C (Figure 1a) [5,9,12,19,20,21,42,43];
- an increase of flow stress with increasing temperature—an anomalous positive temperature dependence of the yield strength (at 600–900 °C) is a characteristic feature of the Ni_3_Al phase and its alloys [1,2,5,11,12,19,21,43];
- a high corrosion resistance in oxygen and carbon enriched atmospheres up to 1100 °C, due to a formation of a continuous surface alumina layer (see Table 1) [5,9,12,19,20,21,43,44,45];
- a high corrosion resistance in organic acids (oxalic and acetic acids), bases (sodium and ammonium hydroxides), and sodium-chloride solution [44,46,47,48,49,50];
- a high fatigue strength resulting from the elimination of stress concentrations on the second phase particles (e.g., carbides) [9,19,21];
- a high creep resistance (which is also affected by a grain size) [9,10,12,19,21,42,51,52];
- an excellent high temperature (above 600 °C) wear resistance [9,12,21,43,44,45,46];
- a relatively low density giving a high strength to weight ratio (Figure 1b) [5,9,12,19,20,21,42,43];
- and recently, catalytic activity in decomposition of various chemical compounds, e.g., methanol, methane, hexane and also sarin and mustard gas and their imitators [22,23,24,25,26,27,28,29,30,31,32,33,34,35].

However, the Ni_3_Al alloys (as well as other intermetallics) also have a number of common drawbacks—mainly related to their low susceptibility to plastic deformation and a high tendency to brittle cracking—that strongly limit their industrial usefulness, especially as components with a minimal linear dimension (a thickness) below 400 µm [53].

**Table 1:** Chemical compositions of Ni_3_Al based alloys selected for commercial applications and for a comparison with conventional high temperature alloys (based on [9,12,19,54,55,56,57,58,59]).

Alloy	Chemical Composition (wt%)										
	Al	Cr	Mo	Zr	B	C	Fe	Ti	W	Si	Ni
IC-50	11.30	**–**	**–**	0.60	0.02	**–**	**–**	**–**	**–**	**–**	balance
IC-221M	8.0	7.70	1.43	1.70	0.008	**–**	**–**	**–**	**–**	**–**	balance
IC-218	8.65	7.87	**–**	0.86	0.02	**–**	**–**	**–**	**–**	**–**	balance
IC-396	7.98	7.72	3.02	0.85	0.005	**–**	**–**	**–**	**–**	**–**	balance
IC-438	8.10	5.23	7.02	0.13	0.005	**–**	**–**	**–**	**–**	**–**	balance
IC-6	7.8 ÷ 8.5	**–**	14.00	**–**	0.03 ÷ 0.15	**–**	**–**	**–**	**–**	**–**	balance ÷ balance
VKNA-1V	8.83	5.58	3.50	0.45	**–**	0.03	**–**	1.54	2.82	**–**	balance
Haynes 214	4.50	16.00	**–**	**–**	**–**	0.03	3.00	**–**	**–**	0.10	balance
FeNiCr (HU)	**–**	18.00	**–**	**–**	**–**	0.55	42.45	**–**	**–**	**–**	balance
Alloy 800	0.40	21.00	**–**	**–**	**–**	0.05	45.50	0.40	**–**	**–**	balance

## 3. Applications of Ni_3_Al-Based Alloys

Over the last twenty years, the results of intensive work on a relationship between a technology, a structure and properties of the Ni_3_Al based alloys have led to the development of a number of engineering alloys with strictly designed compositions (Table 1). Due to their doubtless advantages over the “classic” materials these alloys have found a number successful commercial applications.

**Figure 2:**
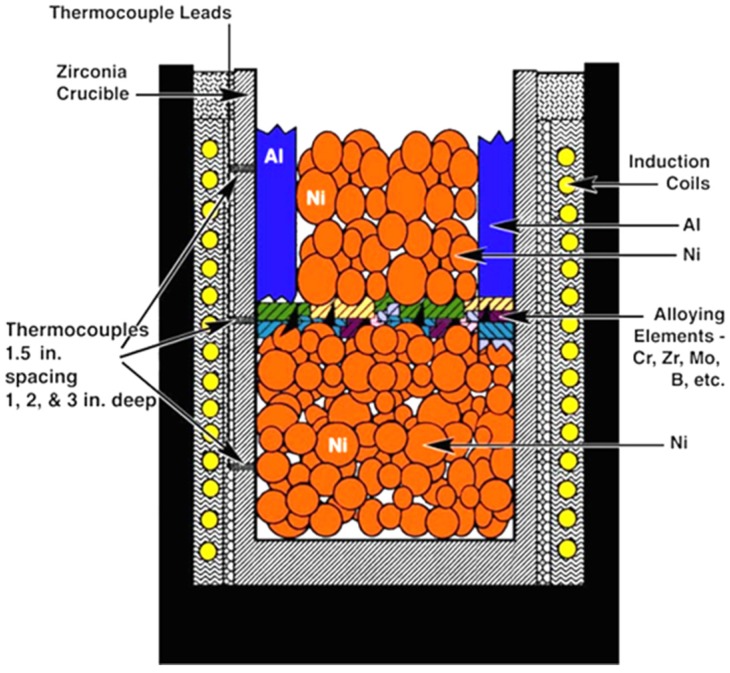
A scheme of furnace loading employed in the Exo-Melt^TM^ process for melting and casting of nickel aluminides [54] (*Courtesy of Oak Ridge National Laboratory, U.S. Department of Energy*).

As a consequence of the conducted research, the main technological problems associated with the production of components made from Ni_3_Al based alloys have been solved (namely, certain difficult aspects of melting, casting and joining technologies). A high aluminum content and a large difference between melting points of constituent elements cause difficulties with maintaining a selected alloy composition or lead to oxidation or porosity of fabricated ingots. In order to minimize these effects, “Exo-melt” process was developed by ORNL in 1996—Figure 2. Due to a special arrangement of particular elements in a crucible, this process uses a heat generated during the exothermic reaction to melt all constituents in a very short time. Moreover, the Exo-Melt process is also beneficial in terms of production costs, giving approximately 50% saving of both time and energy [9,10,54,55,56,60,61]. The Exo-Melt process is employed for melting of nickel aluminides in, e.g.,: Alloy Engineering and Casting Company in Champaign, Illinois; United Defense in Anniston, Alabama; The BiMac Corporation in Dayton, Ohio; and Sandusky International in Sandusky, Ohio.

**Figure 3:**
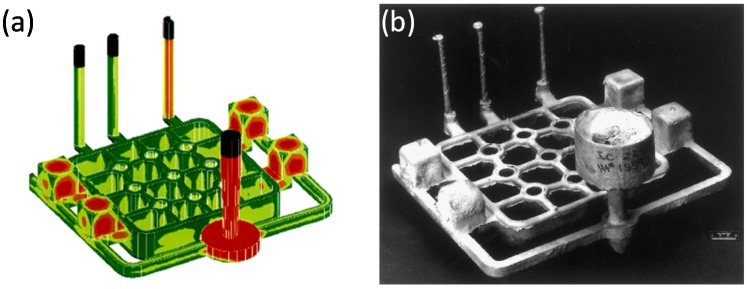
Modeling of the casting process in ProCast software: (**a**) a model [62] (*Courtesy of Oak Ridge National Laboratory, U.S. Department of Energy*); (**b**) a real component (reprinted with permission from Elsevier, 2000 [19]).

Another significant achievement was a development of the casting process using ProCast software (Figure 3a). It should be noted that a casting of Ni_3_Al based alloys is rather difficult due to a low fluidity and shrinkage of the as cast material. However, it was reported that an implementation of ProCast software allows casting of defects-free components with a complex shape (Figure 3b) [19].

**Figure 4:**
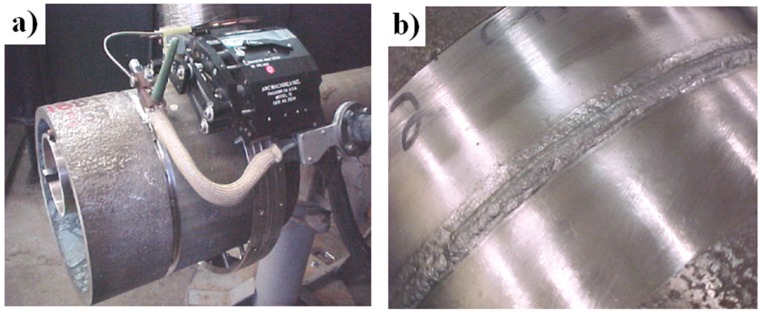
A welding by the TIG method: (**a**) a working setup; (**b**) a weld joins segments of roll made of IC-221 M alloy [54] *(Courtesy of Oak Ridge National Laboratory, U.S. Department of Energy*).

In order to increase a suitability of the Ni_3_Al alloys to industrial demands (a production of elements with complex shapes and an easiness of repair) welding and overlaying welding technologies have also been developed. A lot of works in this field were devoted to IC-221 M alloy and were focused on a selection of both a welding method and a proper binder. It was shown, that Tungsten Inert Gas (TIG) and Metal Inert Gas (MIG) methods (gas shield: 50% Ar and 50% He) allow obtaining high quality joints that are characterized by mechanical properties similar to those of the base material (Figure 4) [54,63,64].

**Figure 5:**
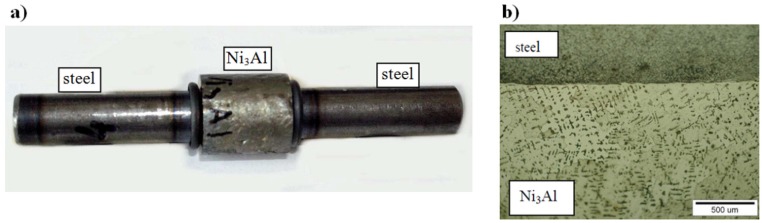
The Ni_3_Al/steel joints obtained by a friction welding technique. (**a**) A macroscopic view and (**b**) a microstructure image [65].

Moreover, Włosiński *et al.* [65] developed an efficient method of joining the Ni_3_Al based alloys with a carbon steel via a friction welding process (Figure 5). Additionally, it was proven that this process is characterized by approximately 5–10 times lower energy consumption as compared to a resistance welding technique.

### 3.1. Applications of Bulk Materials

The properties of Ni_3_Al based intermetallics discussed earlier allow a lot of actual or near the future industrial applications. Some examples are briefly discussed below:

#### 3.1.1. Compressor and Turbine Blades in Aircraft Engines

**Figure 6:**
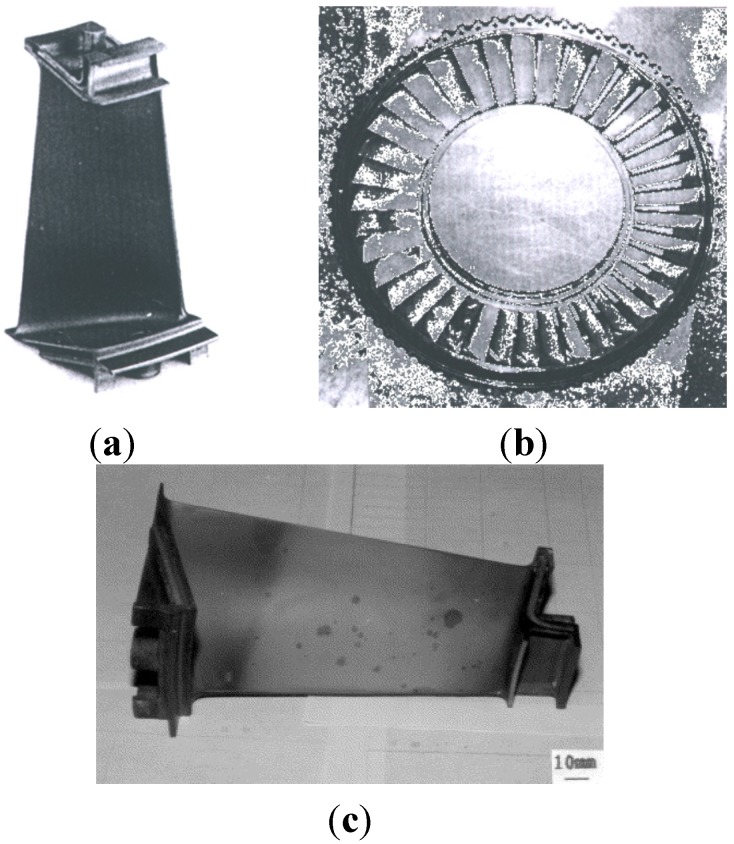
Turbine vanes made of IC 6 Ni_3_Al—based alloy with NiCrAlYSi coating in an advanced air-engine after: (**a**) 25 h engine test [64]; (**b**) 250 h engine test (reprinted with permission from The Minerals, Metals & Materials Society, 1997 [64]); (**c**) 379 h engine test (reprinted with permission from Elsevier, 1999 [67]) (authors [67] stated that the existence of black spots can be related to the internal oxidation of the coating, but the development of this harmful effect was very slow and had not affected the base IC-6 alloy after 379 h of engine tests).

The use of Ni_3_Al as elements of aircraft engines is a classic example, commonly used to demonstrate the potential of future applications. The directionally solidified Ni_3_Al base alloy with commercial name IC6, has been developed for advanced jet-engine turbine blades and vanes operating at the temperature range of 1050–1100 °C (Figure 6). This material with NiCrAlYSi coating is being used for the second stage gas turbine vanes. As reported in references [41,66] stress-rupture strength at 1100 °C/100 h is 100 MPa, *i.e.*, approximately 20 MPa higher than similar Russian BKHA-1Y Ni_3_Al base alloy and American EX-7 alloy, respectively.

The Ni_3_Al based alloys are still regarded as candidates for advanced high temperature structural materials in aerospace applications, e.g., turbine engine components. Investigations and tests are now being conducted on a newly developed Ni_3_Al-based alloys, e.g.,: IC6CX (modified IC6), IC10 and VKNA’s which can be used as materials for advanced aeroengine fan with a service temperature up to 1373 K [41,55,59,64,66,67,68,69,70,71,72,73,74]. As reported in [55,66,73,74] an investigation on replacement of commercially produced nickel alloys such as GS6U, GS26 or ZhS6U with the VKNA-4U are still lasting. An introduction of this Ni_3_Al intermetallic alloy can increase a maximum operation temperature on rotor blades and nozzle guide vane in turbine engines by 50–100 °C, leading to approximately 10% mass reduction and an improvement of heat-resistance. Consequently, it is believed that a service life may increase 2–3 times. It is worth noting, that these materials do not require a strengthening heat treatment. Therefore their manufacturing process is less time consuming and involves a lower consumption of expensive and deficient metals such as tungsten, cobalt, *etc*.

**Figure 7:**
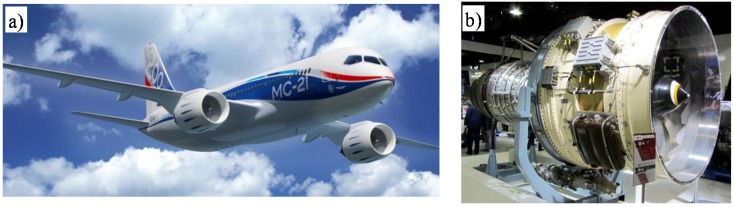
Pictures of: (**a**) Irkut MS-21 twin-jet passenger aircraft [76] and (**b**) its future alternative power plant—PD-14 engine [77].

Actually, aforementioned VKNA’s alloys are tested in prototype PD-14 engine—a next generation turbofan engine which may become one of the alternative power source for the Ilyushin Il-76 and Irkut MS-21 twin-jet passenger aircraft [55,74,75,76,77] (Figure 7).

#### 3.1.2. Turbochargers Rotors in Diesel-Engine Trucks

The Ni_3_Al intermetallics are potential candidates as materials for turbochargers rotors in diesel-engine trucks. As it was reported in [69,78,79], IC-221M alloy (Table 1) may substitute popular IN-713C nickel superalloy that exhibits a worse fatigue strength, a higher density and is more expensive. However, publications on the possible adaptation of Ni_3_Al alloys in this field have not been reported since 2000. Nevertheless, given the ongoing work on aircraft engine applications, their further development cannot be excluded.

#### 3.1.3. Water Turbine Rotors and Water Pumps

**Figure 8:**
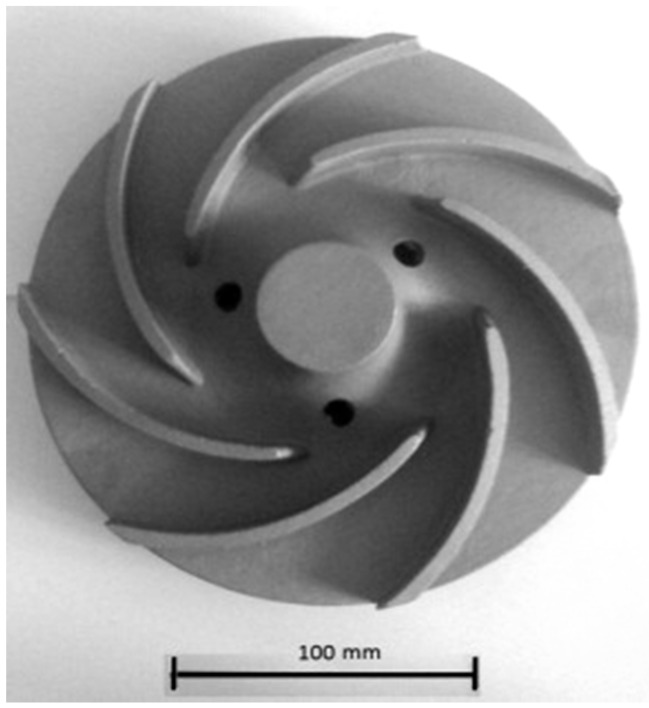
The water turbine rotor made of a Ni_3_Al based alloy (Ni–Al 10.9–Zr 0.22–Cr 6.9–Mo 1.22–Fe 12–B 0.03 (wt%)) [82].

Intermetallic alloys have a much better cavitation and erosion resistance than conventional materials. It is expected that IC-50 alloy (Table 1) may successfully replace actually applied materials [46,80]. It was shown by Zasada *et al.* [81,82] that a water turbine rotor made of a Ni_3_Al based alloy (Ni–Al 10.9–Zr 0.22–Cr 6.9–Mo 1.22–Fe 12–B 0.03 (wt%)) exhibit a definitely longer life time than its counterpart made of stainless steel (Figure 8). Due to a high corrosion resistance, these materials can be used also for working elements in a sea water environment [44].

#### 3.1.4. Car Components

**Figure 9:**
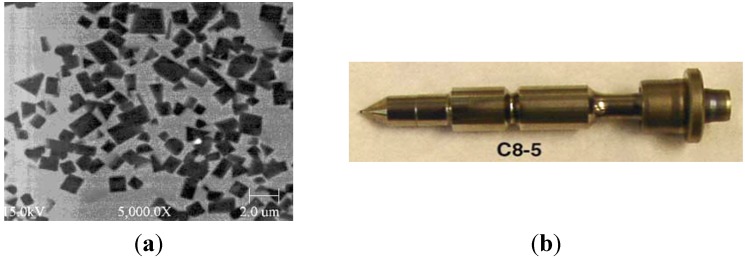
(**a**) A SEM backscattered image of the TiC/Ni_3_Al cermet [85] and (**b**) TiC/Ni_3_Al plunger after tests (Oak Ridge National Laboratory) [86] (*Courtesy of Oak Ridge National Laboratory, U.S. Department of Energy*).

**Figure 10:**
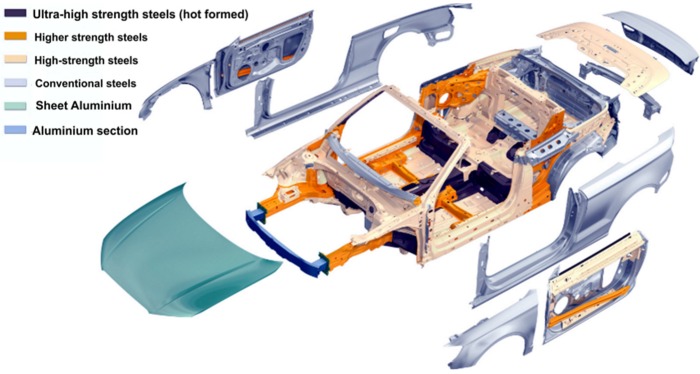
The Audi A3 Cabriolet (2015) materials in the body structure body [92].

- piston rings and valves of internal combustion engines—completely made of a Ni_3_Al based alloy or the Ni_3_Al based composites strengthened by ceramic particles, e.g., Al_2_O_3_, Cr_3_C_2_, Cr_2_O_3_ and SiC [83,84].
- elements of injection systems (e.g., metering plungers) (Figure 9)—an increased pollution emission of diesel engine and a continuous growth of energy efficiency require higher pressures of fuel injection ensuring more precise control of fuel injection. TiC/Ni_3_Al composites with a good wear properties against steel elements are needed for applications where components slide and impact against each other. Figure 9b shows element made of TiC-50 vol% Ni_3_Al which successfully completed the 20-h high pressure (>315 MPa) fuel injection tests [85,86,87].
- automotive body material—works concerned on applications of Ni_3_Al intermetallic alloys to automotive body were recently published [88,89,90,91]. As reported in the papers, this material is lighter and 5 times stronger than stainless steel and exhibits a higher corrosion resistance than currently used automotive materials. Therefore Ni_3_Al alloys can be used not only as automotive body material and also as elements with superior strength or absorbing energy. However, due to its cost, Ni_3_Al intermetallic alloys may only be applied to higher end models, e.g.,: Audi, Mercedes and BMW (Figure 10).

#### 3.1.5. A Steel Industry

**Figure 11:**
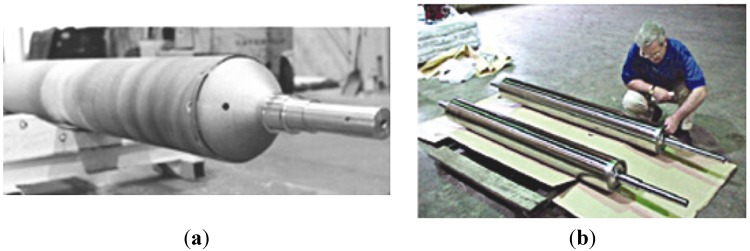
A preparation of transfer rolls in furnaces to: (**a**) austenitization process (a single roll is made of centrifugally casted IC-221M pipe with a diameter of 675 mm and a length of 6.10 m—US Steel) [19]; (**b**) a hydrogenation process (Weirton Steel Corporation) [52].

**Figure 12:**
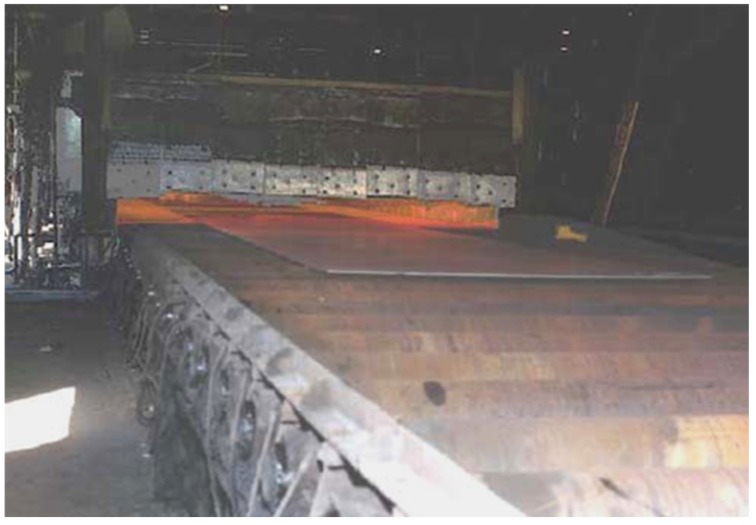
Intermetallic transfer rolls at work—transportation of steel sheets into a heat treatment furnace (Bethlehem Steel) [54] (*Courtesy of Oak Ridge National Laboratory, U.S. Department of Energy*).

Applications of Ni_3_Al alloys in the steel industry was started earliest from other topics and this area is the most popular. A lot of information has been already included in the literature (the most important):

- transfer rolls in furnaces designed for a thermal treatment, carburization and hydrogenation processes; also as rolls in a continuous casting process (Figure 11 and Figure 12) [5,6,7,8,9,10,19,20,21,42,52,54,56,57,61,78,93,94,95].

A replacement of actually used stainless steel by IC-221M alloy allows for significant savings in energy costs by not requiring a water cooling and by extending the working life four to six times over currently used materials [12]. It is estimated that by 2020 the use of IC-221M alloy as transfer rolls material will bring in USA savings of $25 million per year [54,94].

**Figure 13:**
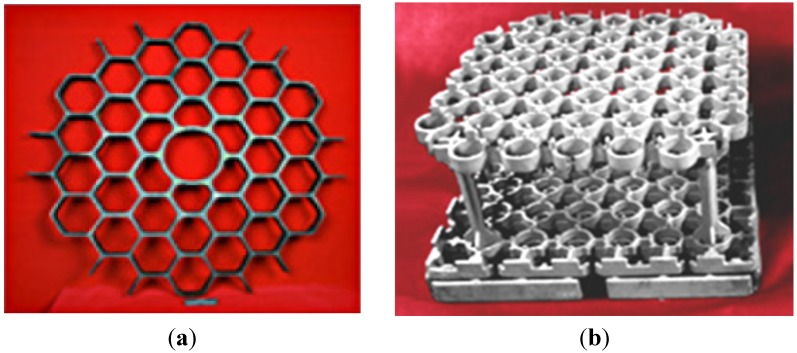
(**a**) A heat treating tray in a steel carburizing furnace (Timken Company [52]) and (**b**) a set of trays (with a total weight of 272 kg) (GM Delphi Saginaw Steering Systems [97]); as casted components made of IC-221M alloy (*Courtesy of Oak Ridge National Laboratory, U.S. Department of Energy*).

**Figure 14:**
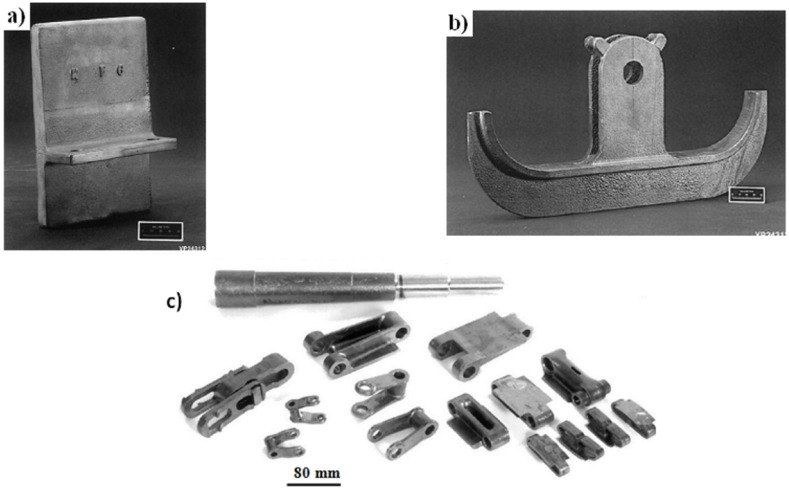
Accessories of heat treatment furnaces: (**a**) a pallet tip [57]; (**b**) a tube hanger [57] and (**c**) belt furnace links made of IC-438 alloy (reprinted with permission from Elsevier, 2000 [19]).

- elements and components of furnaces to heat treatment and carburization processes, e.g., heat-treating trays, tube hangers, link belts, furnace muffles, bolts (Figure 13 and Figure 14) [6,9,10,12,19,57] (*Courtesy of Oak Ridge National Laboratory, U.S. Dept. of Energy*).

A lifetime of trays in heat treatment furnaces (GM Delphi Saginaw Steering Systems) made of traditional materials is only 12–13 months. However, trays made of IC-221M were exploited for at least 3.5 years without showing any signs of damage. It is believed that by 2020 the use of IC-221M alloy as the material for various furnaces components will bring in USA savings of $100 million per year [6,95,96,97].

**Figure 15:**
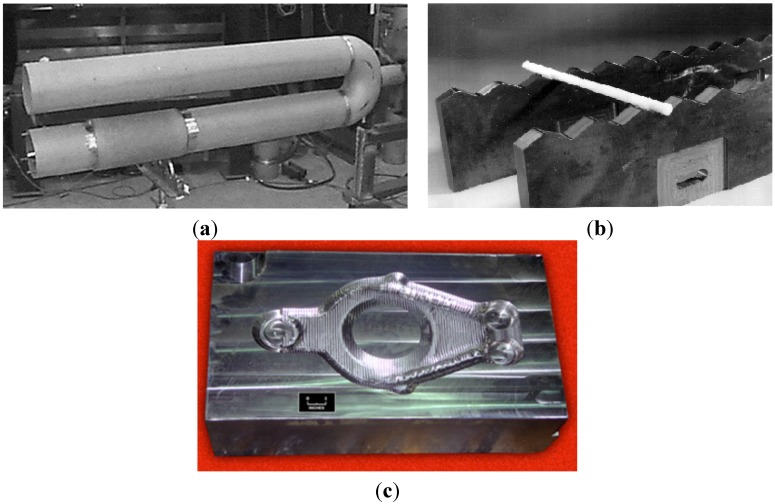
(**a**) The radiant burner tube made of IC-221M alloy (Weirton Steel Corporation) [99]; (**b**) walking-beam furnace rails made of IC-221M Ni_3_Al-based alloy (Rapid Technologies, Newman) [61] and (**c**) die block of IC-221M for mechanical forging hot forging (United Defense LP/Steel Products Division) [101] (*Courtesy of Oak Ridge National Laboratory, U.S. Department of Energy*).

**Figure 16:**
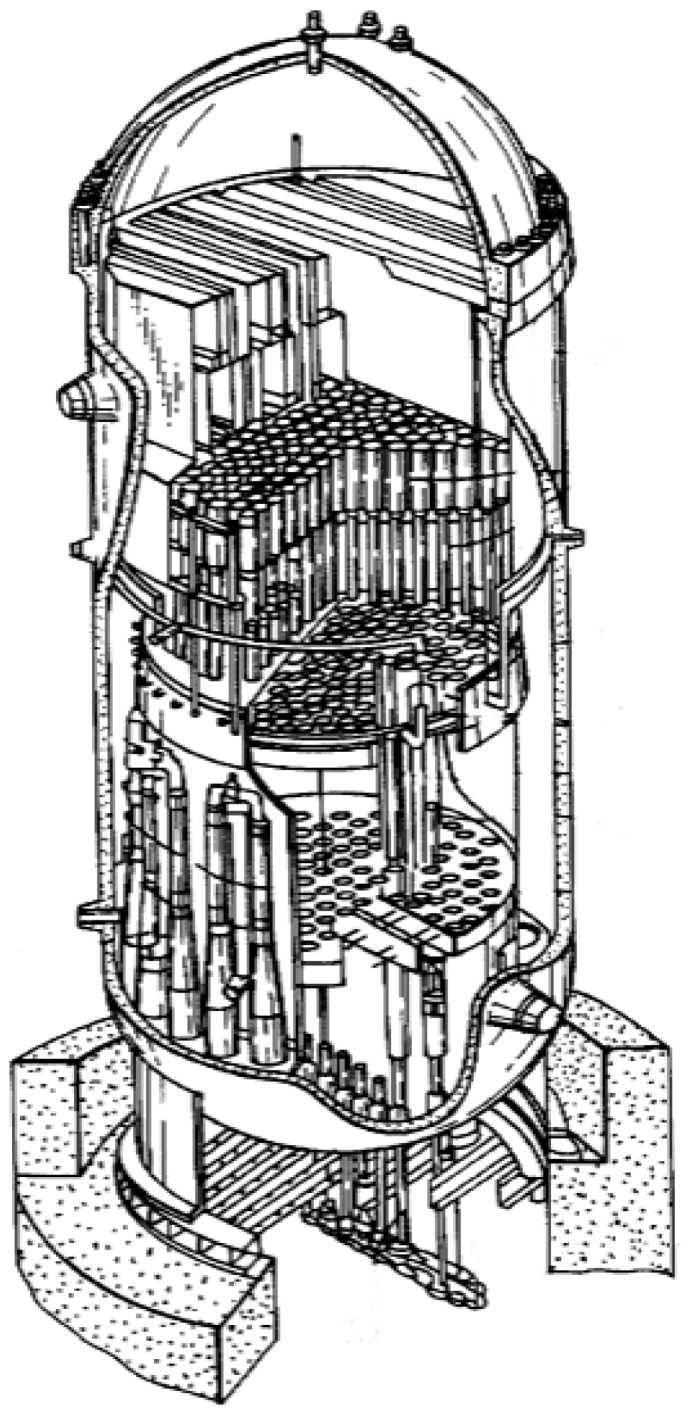
A schematic drawing of a light-water reactor [104].

- centrifugally casted components of radiant-burner-tube set for gas heating devices (Figure 15a) (e.g., Hoskins Manufacturing Company, Weirton Steel Corporation, Sandusky International, Ford Motor Company) [6,19,52].
- rails for walking-beam furnace which are used for heating of steels before a hot forging process (Figure 15b) (e.g., firms: Rapid Technologies, BIMAC Corporations, Cast Masters). The rails supports a moving a processed component from the loading end to the exit and after reaching the set temperature in the range of 1100–1200 °C [1,52,54,61].
- die blocks for closed-die hot forming process (United Defense LP/Steel Products Division, Metallamics) [6,9,10,12,97,98,99,100,101,102]. A higher wear resistance, a higher strength and a resistance to thermal fatigue are the main advantages of Ni_3_Al components, which are taken into consideration in this application. It was reported that dies made of IC-221M alloy exhibit almost 10-times higher durability than that made of HU steel (Figure 15c). Ni_3_Al alloy forging dies was used to successfully forge 100,000 pieces of a part known as a “brake spider” [6,97].

- parts of light-water reactors (e.g., the cladding) having limited irradiation by fast neutrons [103,104,105]. Approximately 30% of electricity in Japan is produced by nuclear power plants—most of them are equipped with light-water reactors (Figure 16).

### 3.2. Applications of Ni_3_Al Thin Foils

Due to special physical and chemical properties associated with a relatively low weight, it is expected that Ni_3_Al intermetallics in the form of thin foils and tapes should significantly contribute to a production of high tech devices of Micro Electro-Mechanical Systems (MEMS) or Microtechnology-based Energy and Chemical Systems (MECS).

**Figure 17:**
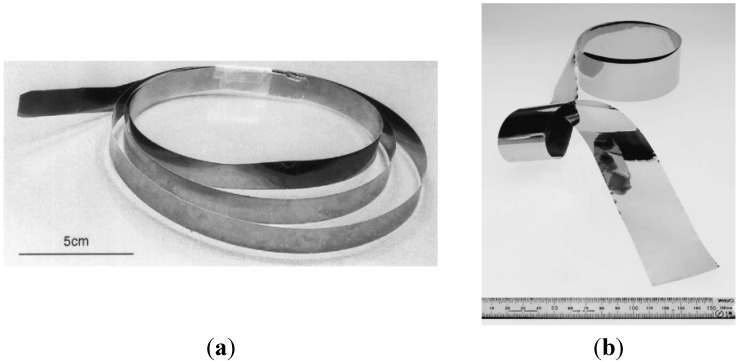
The Ni_3_Al thin foils produced according to technology by NIMS group. (**a**) thickness of 91 µm (reprinted with permission from Elsevier, 2001 [108]); (**b**) thickness of 23 µm (reprinted with permission from Elsevier, 2002 [109]).

**Figure 18:**
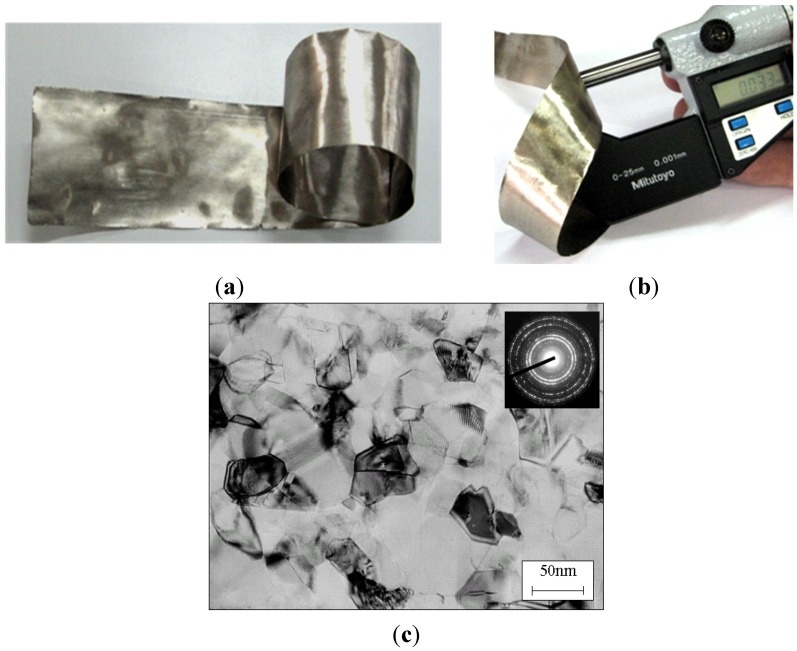
The Ni_3_Al thin foils produced according to technology by MUT group. (**a**) thickness of 80 µm; (**b**) thickness of 33 µm [32] and (**c**) TEM bright field of nanocrystalline Ni_3_Al foils (reprinted with permission from Elsevier, 2006 [115]).

However, the Ni_3_Al alloys have also a few drawbacks—mainly related to their low susceptibility to plastic deformation and a high tendency to brittle cracking—that strongly limit a possibility of industry production of components with thickness below 400 μm [53]. Nevertheless, there are two processing methods already developed in a laboratory scale:

- based on a directional solidification and a cold rolling—proposed by Hirano *et al.* from National Institute for Materials Science NIMS (Japan) with a collaboration of *Ni_3_Al thin foils group* established in 2000, e.g., Oak Ridge National Laboratory and Oregon State University (USA), Max-Planck Institute fur Eisenforschung (Germany), Oregon State University [106,107,108,109,110,111,112,113,114];
- without a costly and time-consuming directional crystallization; based on a controlled deformation of the conventionally casted alloys—proposed by Bojar *et al.* from Military University of Technology (MUT) (Poland). Moreover, this technology was found to give a final product with a higher ductility and a better strength properties (also with nanostructure) than those of Ni_3_Al foils produced by Hirano group (Figure 17 and Figure 18) [115,116,117,118,119,120].

It is expected that the Ni_3_Al thin foils will be soon applied as a components of high-tech devices. These potential applications include, e.g., heat exchangers, microreactors, catalysts, intermetallic laminate, microactuators, high stiffness systems or even components of rocket engines [44,121,122,123,124,125,126,127,128,129,130].

Additionally, their catalytic properties may find an application in air purification systems from chemical and biological toxic agents or in a decomposition of hydrocarbons for hydrogen production [23,24,25,26,29,30,31,32,33,34,35,36,131].

**Figure 19:**
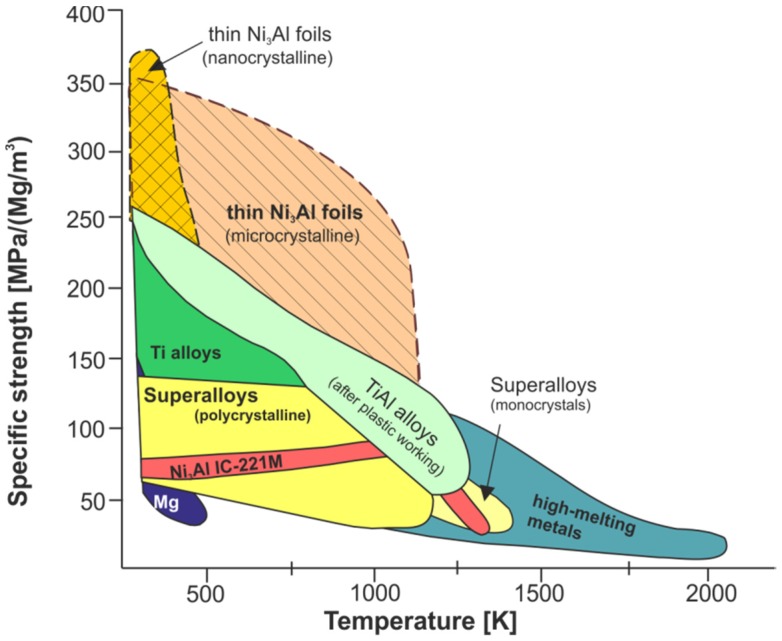
A diagram of specific strength *vs.* temperature of modern structural materials and Ni_3_Al thin foils (based on [1,2,3,4] and [115,116,117,118]).

The MEMS or the MECS types of systems are designed to provide the integration of mechanical (e.g., actuators) and electronic (e.g., the sensor, a microprocessor) components resistant to environmental, giving the possibility to fabricate a device which plays both control and executive functions (Figure 19).

The MEMS technology has already found, or will find in the near future, applications in the following research and production areas:

- fabrication of microsensors of, e.g., acceleration, pressure, flow (e.g., to remote respiratory monitoring system, monitoring the levels of chemical contaminants) and gyroscopes,
- manufacturing of microchips: gears, motors, actuators and so on [122,123,124,125,126,127,128,129,130].

On the other hand, MECS is a relatively new research direction, narrowing the problem of micro-systems issues to a heat and mass transport and to processes occurring in liquids. This research direction has been started by Oregon State University, who works with a research and production centers, e.g.: Defense Advanced Research Projects Agency (DARPA), National Science Foundation (NSF) or Pacific Northwest National Laboratory (PNNL). The MECS technology is designed for applications in, e.g., micropumps, thermal evaporators, fuel cells, chemical reactors, cooling system components or heat exchangers [123,127,128,129].

An extensive development of microsystems requires the use of materials with high strength and special physicochemical properties. A comparison of materials that are actually applied in MEMS devices was shown by Spearing [121] (Table 2). A specific stiffness and a specific strength were recognized as the most important parameters of MEMS designed materials.

**Table 2:** Strength of materials that are actually applied in MEMS devices (based on [121] and [115,116,117,118]).

Material	Density (g/cm^3^)	Young Modulus (GPa)	Tensile Strength (MPa)	Specific Stiffness (GN/kg·m)	Specific Strength (MN/kg·m)
Silicon ^1^	2.33	129–187	4000	55–80	1.7
Silica ^1^	2.20	73	1000	33	0.45
Nickel ^1^	8.90	207	500	23	0.06
Aluminum ^1^	2.71	69	300	25	0.11
Alumina ^1^	3.97	393	2000	99	0.50
Silicon carbide ^1^	3.30	430	2000	130	0.30
Diamond ^1^	3.51	1035	1000	295	0.28
Ni_3_Al (micro) ^2^	7.5	200	2300	26	0.31
Ni_3_Al (nano) ^2^	7.5	200	2900	26	0.39

^1^ based on [121]; ^2^ based on [115,116,117,118].

The Ni_3_Al intermetallics as compared to materials currently used for mechanical components of MEMS devices (Table 2), exhibit not only a high strength but also a relatively high ductility and fracture toughness. These properties, combined with a high oxidation and corrosion resistance, as well as high working temperature (up to 1300 °C) make them an attractive material for MEMS and MECS applications. As reported by Burns *et al.* [122], high thermal stability of the Ni_3_Al intermetallic phase and alloys based on this phase makes them ideal candidates for MEMS works in high-temperature environments.

**Figure 20:**
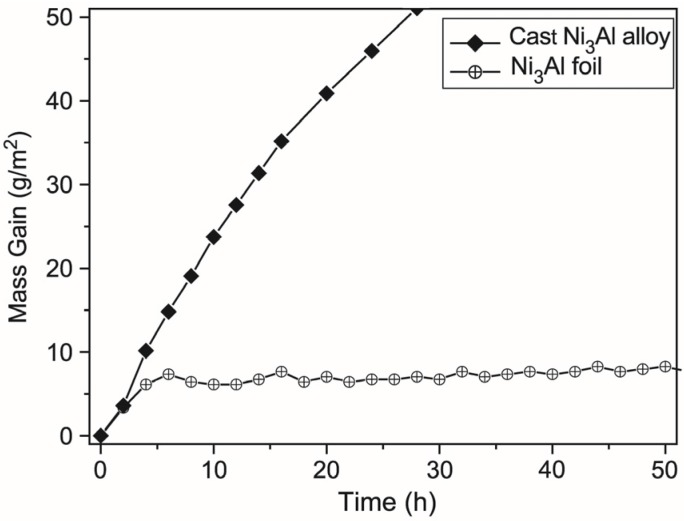
Cyclic oxidation curves at 1000 °C up to 50 h (2 h/cycle) for a cast Ni_3_Al alloy and a cold-rolled Ni_3_Al foil (reprinted with permission from Elsevier, 2004 [113]).

A research conducted within the aforementioned “Ni_3_Al thin foils group” confirmed the high performance characteristics of this type of material—including its high resistance to oxidation. Kim *et al.* [113] shown that the oxidation resistance (measured as a mass gain per unit area) at 1000 °C of the Ni_3_Al thin foil is much better than the same alloy but in the as cast condition (Figure 20). The resulting change in mass of the as cast sample after five hours of the annealing was the same as for thin foil annealed for 50 h. Additionally, the weight of the foil was stabilized its after 5 h of oxidation while the weight of the cast sample was continuously increased. It is worth noting that the oxidation resistance becomes an especially important feature, when a thickness of component is lowered (as in the case of microsystems).

Applications of Ni_3_Al foils and tapes for structural (load-bearing structure, plating), functional and multifunctional components are worked out. Beside of studies on fabrication and processing of the foils, a research is also carried out on joining technologies [131,132,133,134,135,136] and methods of a forming them into a “honeycomb” structures [32,111,134,135]. Mentioned properties of Ni_3_Al thin foils predispose them to applications in:

#### 3.2.1. Structure with a Highly Developed Active Surface, e.g., “Honeycombs”, Heat Exchangers, Catalysts and Also Filters

In gas turbine applications, one important component made of thin foil is a turbine seal ring assembly that controls the turbine tip clearance for improving thermal efficiency. The seal ring assembly is typically constructed out of honeycomb seals brazed onto a superalloy casting. The traditional alloys used for honeycomb samples are chromia formers, e.g., nickel-based superalloys. Thin foils made of oxidation-resistant alloys including stainless steels and nickel-base alloys have been extensively evaluated as high-temperature heat exchanger in microturbines. These heat exchangers used for preheating the incoming air for combustion can significantly growth effectiveness of microturbine.

**Figure 21:**
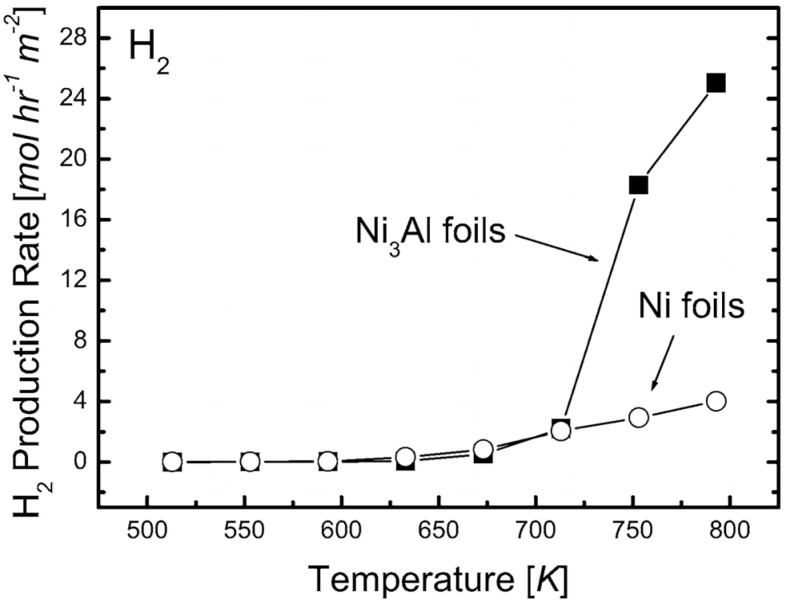
Comparison of production rates of H_2_ in methanol decomposition of Ni_3_Al foils and Ni foils (reprinted with permission from Elsevier, 2006 [25]).

Catalysts for a decomposition of hydrocarbons (e.g., methanol, methane, hexane) in terms of the “production” of hydrogen (National Institute for Materials Science, Military University of Technology, Tomsk State University). Results presented in [22,23,24,25,26,27,28,29,30,31,32,33,34,35] clearly points toward a superiority of these materials over conventionally used nickel catalysts (Figure 21).

**Figure 22:**
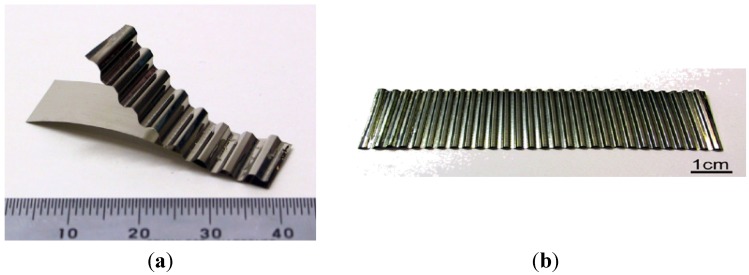
A blank of the honeycomb structure (**a**) the Ni_3_Al strip [111]; and (**b**) a manufactured blank of an automotive catalyst [112].

**Figure 23:**
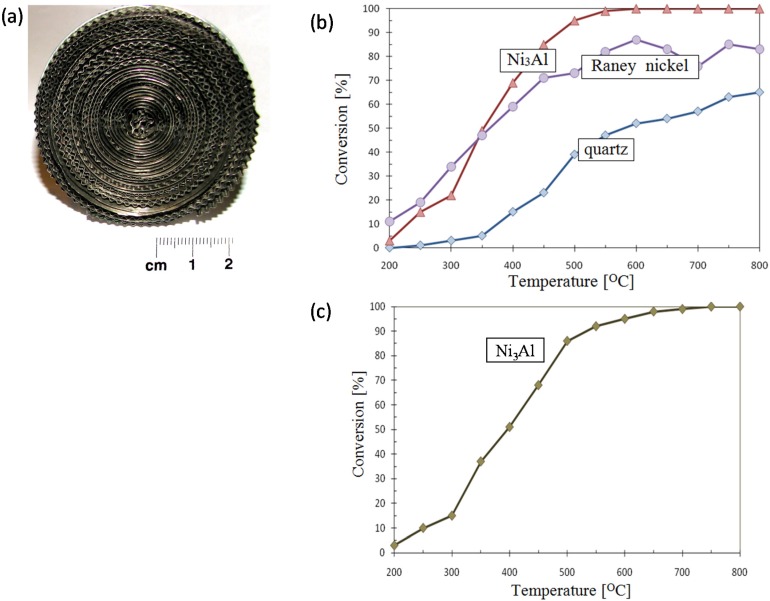
An example of honeycomb structure made of the Ni_3_Al thin foil (**a**) [32] and a catalytic activity of the Ni_3_Al foil (a comparison to Raney nickel and quartz) upon a decomposition reaction of: (**b**) hexane, (**c**) gas mustard imitator [28].

- catalytic converter (NIMS, Nippon Cross Rolling Corporation, Nippon Steel Technoresearch Corporation)—Ni_3_Al thin foils in the form of a flat honeycomb structure with narrow gaps (Figure 22) [111].
- thermocatalytic air purification systems—the main function of this type of devices is to remove all kinds of toxic chemicals including warfare agents (e.g.,: sarin, mustard gas—Figure 23) and dangerous biological agents from the air. In contrast to conventional filtering devices (where after the hazardous substances are retained in the filters causing the need for their frequent replacement) designed device using thermocatalytic processes, remove them completely. Therefore, the “hot filter” works more efficiently and much faster as compared to conventional filtration systems [32,131].

#### 3.2.2. Electronic Equipment

Specific properties of the Ni_3_Al intermetallics are also attractive for electronic industry. A high specific strength combined with a high operating temperature (at least 1000 °C) and a high thermal conductivity make them attractive to a wide range of applications. This field includes, e.g., ancillary components such as IC substrates. This material can be also regarded as a model system of a template for a production of well-ordered Al_2_O_3_ surface layer. This kind of surface layer may be used in research works on various surface phenomena.

- electronic devices on a substrate made of IC-50 alloy (see Table 1)

**Figure 24:**
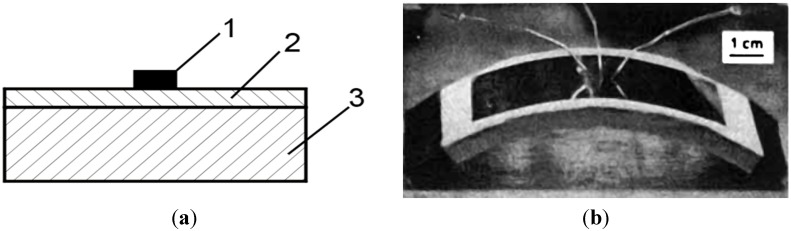
(**a**) A schematic diagram of the idea of integrated circuit on a Ni_3_Al substrate: 1—electronic component, 2—isolating alumina layer, 3—Ni_3_Al substrate (based on [137]); (**b**) TFT liquid crystal displays on a metallic substrate upon a bending test (a curvature radius of 8.25 cm) [139].

The Ni_3_Al intermetallics have a sufficiently high aluminum content to create a surface continuous alumina layer which possess a much better adhesive resistance and thermal shock resistance than the chromia layer. The thickness of this layer is large enough to allow placing a chip without contact with the metallic layer (Figure 24a). A higher thermal conductivity of such a substrate allows for a more efficient heat dissipation (50–100 W/cm^2^), and thus, a higher operating temperature of the system and its longer service life. The Ni_3_Al thin foils are elastic, they have a higher thermal conductivity coefficient and their excellent heat resistance allows for high temperature applications [137].

- liquid crystal displays

The Ni_3_Al intermetallic foils with a thickness of 25–200 μm are considered as a potential substitutes of the glass substrates in thin-film transistors (TFT) displays (Figure 24b). These displays have a lower weight, a better flexibility (e.g., withstand a bending to the curvature radius of approximately 10 cm) and a much higher impact resistance (they are not damaged when falling from a few meters) [138,139,140].

**Figure 25:**
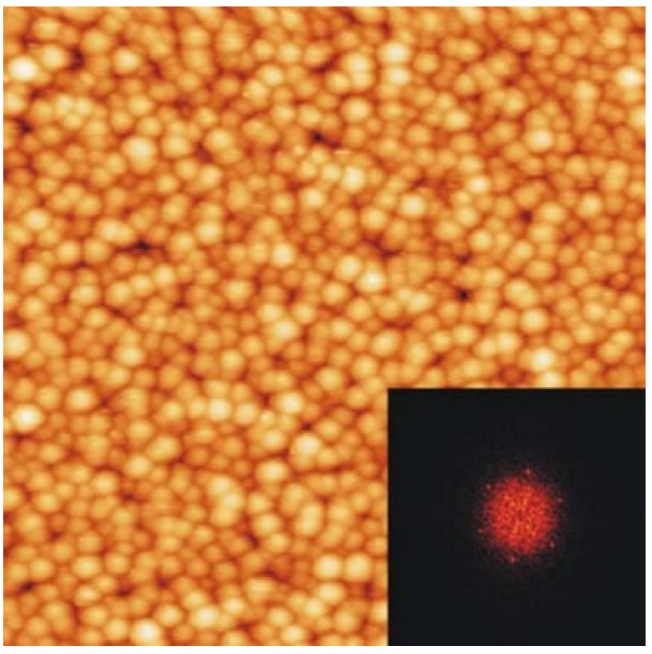
The STM image of Fe clusters deposited on Al_2_O_3_/Ni_3_Al(111) (the FFT shown in the inset evidences a hexagonal arrangement of the iron clusters with a nearest neighbor distance of 24 Å) (reprinted with permission from Elsevier, 2006 [150]).

The Ni_3_Al alloys surfaces, including the Ni_3_Al(111) and Ni_3_Al(001) surfaces, have been investigated experimentally [141,142,143] and theoretically [144,145,146], and are often used as a substrate in studies of a variety of different surface phenomena such metal thin film growth (e.g., Pb/Ni_3_Al(111) [143]), oxidation (e.g., Al_2_O_3_/Ni_3_Al(111) [147], Al_2_O_3_/NiAl(110) [148]) and the formation of more complex systems like CuPc/Al_2_O_3_/Ni_3_Al(111) [149] or Fe/Al_2_O_3_/Ni_3_Al(111) (Figure 25) [150]. The Ni_3_Al can be seen as a model system for use as a template for a well-ordered Al_2_O_3_ surface [142,147], which is suitable for studies with several surface sensitive investigation methods requiring electric conductivity.

#### 3.2.3. Mechanical Systems and Other

Mechanical systems are an almost perfect example of using the Ni_3_Al intermetallics. The most popular fields of application in this area include:

- micro gears and micromotors

**Figure 26:**
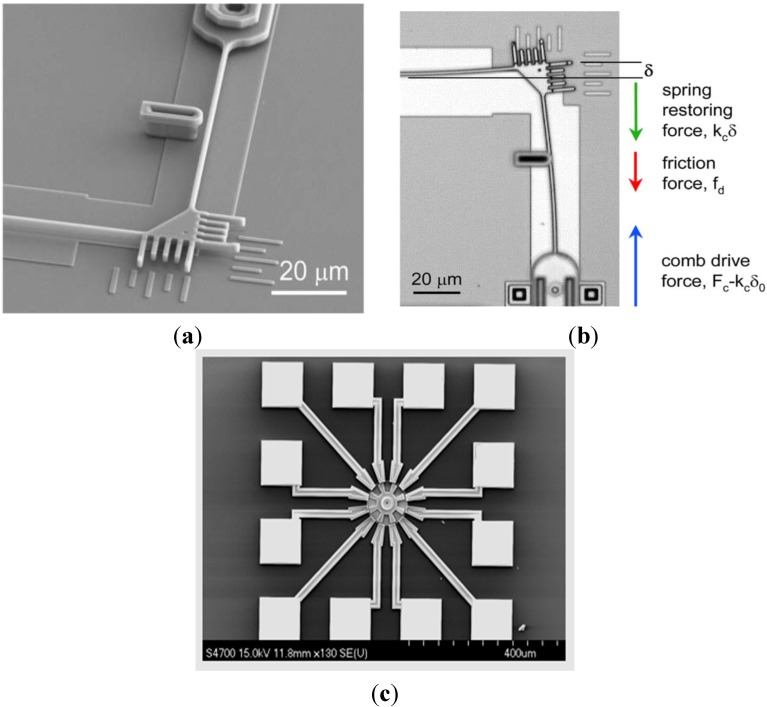
Micromachines made in the MEMS technology: (**a**) SEM microphotographs of the beam and anchored post of sidewall tribometer from electrostatic actuators and (**b**) a top view during data collection in the optical microscope (reprinted with permission from Elsevier, 2003 [127]); (**c**) a surface micromachined electro-statically-actuated micromotor fabricated by the MNX (MEMS and Nanotechnology Exchange), this device is an example of MEMS-based microactuator (*the following picture of MEMS and Nanotechnology Exchange is provided courtesy of Dr. Michael Huff of the MEMS and Nanotechnology Exchange, see: http://www.mems-exchange.org at the Corporation for National Research Initiatives*) [126].

**Figure 27:**
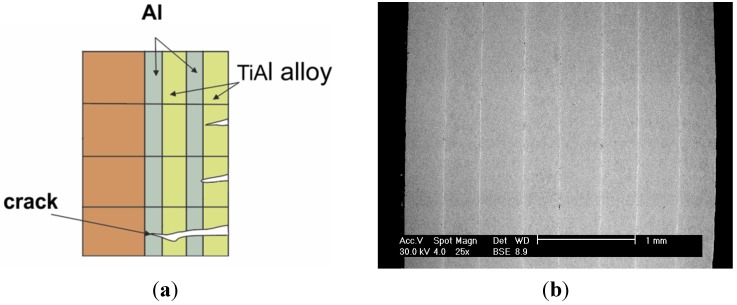

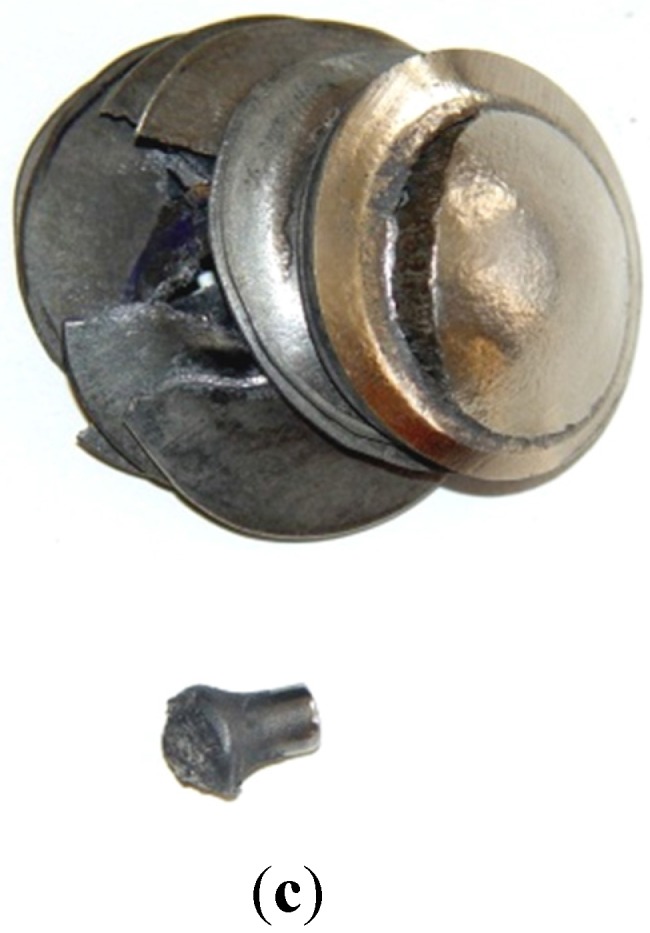
(**a**) A scheme of metallic/intermetallic laminate composed of aluminum alloy and Ti–Al intermetallic alloy (based on [151]), and (**b**) a SEM microphotograph of Ni_3_Al multilayer material (eight Ni_3_Al plates obtained by explosive welding) [136]; (**c**) a pack of a Ni_3_Al (Zr, B) plates (packed loosely) after a shooting test with 7.63 mm caliber bullet (kbk AK 47) with a view of deformed bullet core [118].

**Figure 28:**
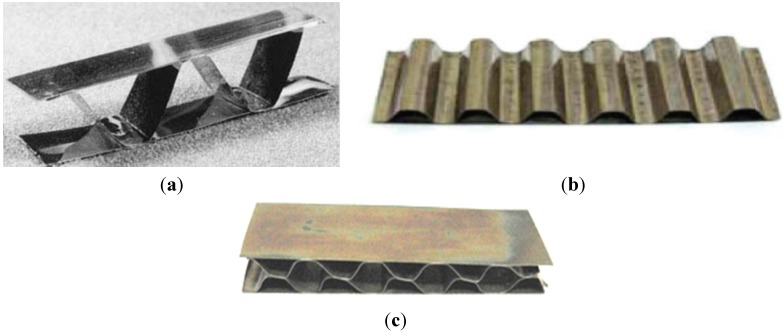
An example of honeycomb structure made of the Ni_3_Al thin foil fabricated by: (**a**) a laser welding (reprinted with permission from The Minerals, Metals & Materials Society, 2001 [152]); (**b**) a resistance welding [32]; (**c**) a soldering [32].

A large fragility of silicon, which is the most popular material used in mechanical micro-components and micro-gears, limits application possibilities of these elements. Therefore, there is a chance for the Ni_3_Al foils to fill this gap [38,53,64,112,122,124,125,126,127,128,129,130]. Due to the high specific strength combined with a good resistance to oxidation, erosion and abrasive wear it is planned to use the Ni_3_Al foils in following applications: actuators, a “platform” of pressure microsensors and the acceleration microsensors (Figure 26). In the case of pressure microsensors a flexibility of the Ni_3_Al foil gives a possibility of production a version with the substrate susceptible to significant shape changes:

- armor and ballistic shields (Figure 27) (DARPA, University of California) fabricated as a metallic/intermetallic laminate (MIL). Such a solution allows for the combination of high stiffness and strength with a high resistance to rupture and fragmentation [151].
- honeycomb structures—a LFB (lighter, faster, better) is one of the most important criteria in modern designing (Figure 28). An extremely high specific strength (Figure 19) combined with a high operating temperature (900–1000 °C) and corrosion resistance makes the Ni_3_Al thin foils a great structural materials.

## 4. Summary

Results of the presented literature survey clearly confirm the superiority of mechanical properties of Ni_3_Al based alloy over actually applied heat and creep resistant materials. Oak Ridge National Laboratory is the world leader in basic research, development and commercial implementation of Ni_3_Al intermetallics. This institution has been collaborating on an introduction of Ni_3_Al alloys into metallurgy and heat treatment industries as components of furnaces for hot working and thermo chemical treatments, e.g., transfer rolls, trays, plungers, dies, *etc.* On the other hand, Beijing Institute of Aeronautical Materials has achieved promising results in the field of Ni_3_Al alloys applications related to the aviation industry. This institute has developed and introduced directionally solidified IC10 alloy for the turbine vanes and is still developing this technology. In the field of advanced aeroengines applications, research is also conducted in Russia resulting with a development of the VKNA alloys. Tests are also carried out on a new engine PD-14, which is intended for usage in Ilyushin Ił-76 and Irkut MS-21 aircrafts.

A growing interest in Ni_3_Al intermetallics in the form of thin foils, with a superior specific strength, a high environmental resistance and high catalytic activity, is observed. Moreover, a development of composite materials with Ni_3_Al based alloys as a matrix hardened by e.g., TiC, ZrO_2_, WC, SiC and graphene is also observed.

The MEMS or MECS devices are a highly perspective applications of foils/strips Ni_3_Al based alloys. Their specific properties seem to be especially useful in the production of microsensors, microsystems of chemical separators, heat exchangers and heat micropumps.

However, it is worth noting, according to Szafrik [153], that an implementation of new solutions can affect constructors’ conservative approach to materials designing, resulting in an aversion to used intermetallics instead of materials with theoretically higher reliability. It is highly probable that attempts to an introduction of “classic”—bulk Ni_3_Al alloys will give way to a their low dimensional forms (e.g., foils or strips), also including their nanostructural counterparts, which are a new, strongly growing, trend.
